# Evaluation of Four Different Systems for Extraction of RNA from Stool Suspensions Using MS-2 Coliphage as an Exogenous Control for RT-PCR Inhibition

**DOI:** 10.1371/journal.pone.0039455

**Published:** 2012-07-16

**Authors:** Lester M. Shulman, Musa Hindiyeh, Khitam Muhsen, Dani Cohen, Ella Mendelson, Danit Sofer

**Affiliations:** 1 Central Virology Laboratory, Public Health Services, Israel Ministry of Health, Sheba Medical Center, Tel Hashomer, Israel; 2 Department of Epidemiology and Preventive Medicine, School of Public Health, Sackler Faculty of Medicine, Tel Aviv University, Tel Aviv, Israel; University of Houston, United States of America

## Abstract

Knowing when, and to what extent co-extracted inhibitors interfere with molecular RNA diagnostic assays is of utmost importance. The QIAamp Viral RNA Mini Kit (A); MagNA Pure LC2.0 Automatic extractor (B); KingFisher (C); and NucliSENS EasyMag (D) RNA extraction systems were evaluated for extraction efficiency and co-purification of inhibitors from stool suspensions. Real-Time Reverse Transcriptase Polymerase Chain Reaction (rRT-PCR) of MS-2 coliphage spiked into each system’s lysis buffer served as an external control for both. Cycle thresholds (Cts) of the MS2 were determined for RNA extracted from stool suspensions containing unknown (n = 93) or varying amounts of inhibitors (n = 92). Stool suspensions from the latter group were also used to determine whether MS-2 and enterovirus rRT-PCR inhibitions were correlated. Specifically 23 RNA extracts from stool suspensions were spiked with enterovirus RNA after extraction and 13 of these stool suspension were spiked with intact enterovirus before extraction. MS2 rRT-PCR inhibition varied for RNAs extracted by the different systems. Inhibition was noted in 12 (13.0%), 26 (28.3%), 7 (7.6%), and 7 (7.6%) of the first 93 RNA extracts, and 58 (63.0%), 55 (59.8%), 37 (40.2%) and 30 (32.6%) of the second 92 extracts for A, B, C, and D, respectively. Furthermore, enterovirus rRT-PCR inhibition correlated with MS2 rRT-PCR inhibition for added enterovirus RNA or virus particles. In conclusion, rRT-PCR for MS-2 RNA is a good predictor of inhibition of enterovirus RNA extracted from stool suspensions. EasyMag performed the best, however all four extraction methods were suitable provided that external controls identified problematic samples.

## Introduction

Diagnosis of enteric viral infections by Real Time reverse transcription - polymerase chain reaction (rRT-PCR) of RNA extracted from stool suspensions is complementing and increasingly replacing diagnosis based on viral isolation and characterization in tissue culture [1], [2]. However, interpretation of results is not always straightforward. The sensitivity of the rRT-PCR is negatively impacted, by compounds present in the clinical sample that may partially or completely inhibit the RT and/or PCR chemistries [3], [4], [5], [6], [7]. Potential inhibitors that might be incompletely removed from stool suspensions during RNA extraction include hemoglobin, immunoglobulins, bilirubin, triglycerides, complex polysaccharides, organic and phenolic compounds, glycogen, fats, and metabolic products especially those from pathological conditions, bacteria, vegetables, medications, anticoagulants, and drugs or alcohol [4], [6], [8], [9], [10], [11]. Adding to the list of endogenous rRT-PCR inhibitors are exogenous inhibitors from extraction protocols such as detergents, chelating compounds and guanidinium HCl [9].

The presence of inhibitory compounds in the extracted RNA and the extent of inhibition can be determined by semi-quantitative rRT-PCR of an RNA template that is present in the sample, added to it prior to extraction, or introduced into the rRT-PCR mix. Natural or modified, encapsulated coliphage MS2 RNA is one source of protected RNA that has been used as a non-competitive external RNA template control [5], [12], [13], [14].

The efficiency of removing inhibitors in patient samples may be related to the intrinsic properties of the method used to extract the RNA [15]. In this study we compared four commercial RNA extraction systems efficiencies for RNA isolation and removal of inhibitors in stool samples. The extraction systems were: QIAamp Viral RNA Mini Kit: manual extraction using silica columns (QIAGEN Inc, Valencia, CA, USA); MagNA Pure LC2.0 Automatic extractor with MagNA Pure LC Total Nucleic Acid Isolation Kit–High Performance: automatic RNA extraction using magnetic beads (Roche Diagnostics, IN, USA); KingFisher (Thermo Electron Corporation, Waltham, MA, USA) semi-automatic extraction using magnetic beads of Ambion MagMAX Viral RNA Isolation kit (Ambion, Inc, Austin, Tx, USA); NucliSENS easyMag (bioMerieux, Marcy l’Etoile, France): semi-automatic extractor using magnetic beads of easyMag extraction kit. In addition, the effectiveness of MS2 as an exogenous template control for measuring inhibitors co-extracted with RNA from stool suspensions and its relevance for validation of enterovirus diagnostic rRT-PCR suspensions is discussed.

## Results

### Comparison of Four RNA Extraction Procedures for Recovery of MS2 RNA from Stool Suspensions

Ninety-three stool suspensions were extracted by the four extraction systems after spiking the lysis buffer of each with MS2 that yielded similar final MS2 concentrations. Any contribution from endogenous MS2 RNA was ruled out since no MS2 RNA was amplified from any of these 93 stool suspensions upon extraction with protocol D when exogenous MS2 was omitted from the lysis buffer. Results for each extraction procedure by assigned levels of inhibition are shown in Table 1. Individual values are shown in Fig. S1. The proportion of samples with inhibitors varied among the extraction methods. Of the 93 RNA extracts, 12 (13.0%), 26 (28.3%), 7 (7.6%), and 7 (7.6%) extracted by protocols A, B, C, and D, respectively, contained inhibitors of MS2 rRT-PCR. Median interquartile range (IQR) reduction in Cts for protocols A, B, C and D were 2.4 (0 to 34), 1.9 (0 to 33), 0.58 (0 to 29), and 0.26 (0 to 7), respectively. Finally, the number of samples with an inhibition >6 Ct were 6 (6.5%), 3 (6.4%), 2 (2.2%), and 1 (1.1%) for Extraction Protocols A through D, respectively. The Friedman Test indicated that there was a statistically significant difference in co-extraction of inhibitors of MS2 rRT-PCR among the extraction protocols used, *P*<0.001.

**Table 1:** Numbers of RNA samples with different levels of inhibition of MS2 rRT-PCR by extraction protocol.

Experiment 1								
Inhibition	A^(a)^		B^(a)^		C^(a)^		D^(a)^	
Ct	No.	%	No.	%	No.	%	No.	%
0	81	87.1	67	72.0	85	91.4	86	92.5
1 to 3	5	5.4	9	9.7	5	5.4	6	6.5
4 to 6	1	1.1	11	11.8	1	1.1	0	0.0
7 to 9	0	0.0	3	3.4	0	0.0	1	1.1
≥10	6	6.5	3	3.4	2	2.2	0	0.0
**Friedman test**								*p = *
mean	2.08		1.82		0.58		0.26	**<0.001**
mean ranks	2.49		2.79		2.38		2.34	**<0.001**
**Wilcoxon with Bonferroni correction^(b)^**								
	*p = *	B	C	D				
	A	0.146	0.039	0.020				
	B		**<0.001**	**<0.001**				
	C			0.844				
**Experiment 2**								
**Inhibition**	**A^(a)^**		**B^(a)^**		**C^(a)^**		**D^(a)^**	
**Ct**	**No.**	**%**	**No.**	**%**	**No.**	**%**	**No.**	**%**
0	34	37.0	37	40.2	55	59.8	62	67.4
1 to 3	9	9.8	14	15.2	13	14.1	21	22.8
4 to 6	5	5.4	12	13.0	10	10.9	7	7.6
7 to 9	7	7.6	9	9.8	3	3.3	1	1.1
≥10	37	40.2	20	21.7	11	12.0	1	1.1
**Friedman test**								*p = *
mean	10.51		6.89		4.07		1.29	**<0.001**
mean ranks	3.11		2.75		2.21		1.93	**<0.001**
**Wilcoxon with Bonferroni correction^(b)^**								
	*p = *	B	C	D				
	A	**<0.001**	**<0.001**	**<0.001**				
	B		**<0.001**	**<0.001**				
	C			**0.001**				

a Equal amounts of stool suspensions chosen randomly from among samples sent to the laboratory from patients with acute gastroenteritis were extracted by four protocols: (A) QIAgen, (B) magNA Pure, (C) KingFisher, and (D) easyMag as described in Methods.

b Bonferroni correction sets the significance level to *P*<0.008.

### Comparison of Four RNA Extraction Procedures on Archived Stool Suspensions with Inhibitors

**Figure 1:**
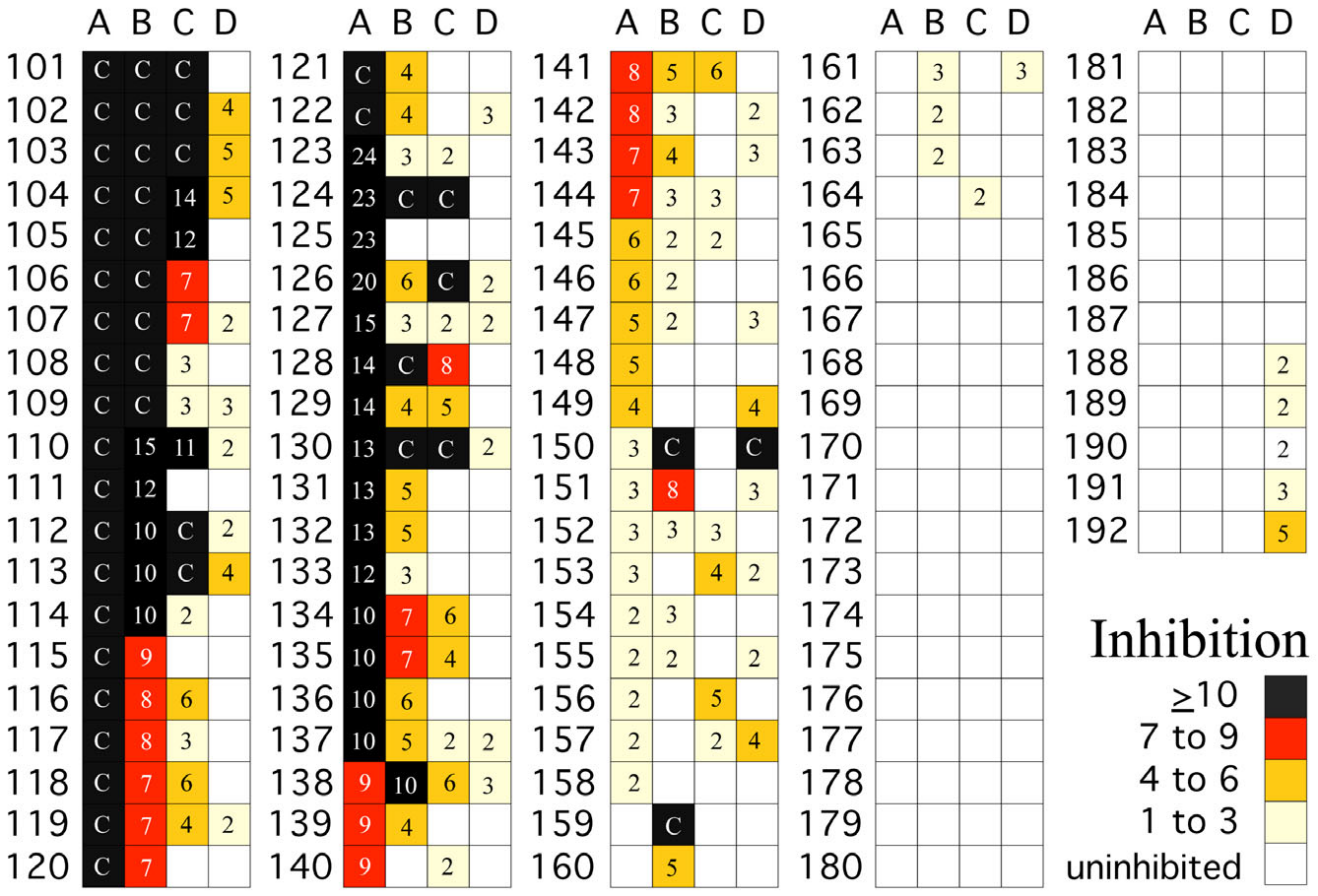
MS2 rRT-PCR inhibition in RNA extracted from stool samples using four different RNA extraction protocols. Equal amounts of stool suspensions prescreened by Protocol A were extracted by four protocols: (A) QIAgen, (B) MagNA Pure, (C) KingFisher, and (D) easyMag as described in methods. MS2 coliphage calculated to give 27 Ct by rRT-PCR was added to the extraction buffer. rRT-PCR values for MS2 in RNA extracted from buffer controls were subtracted from the values for MS2 in RNA extracted from stool suspensions. These differences, the number of Cts of inhibition, are shown in the boxes to the right of the sample numbers. An empty white box indicates that there was no inhibition. Negative values were set to 0 and the maximum values for inhibition “C” were capped at 29 Cts. Samples with inhibition ≥10, 6 to 9, 3 to 6, and 1 to 3 CT are indicated by the colors of the boxes: black, red, tan, and light yellow, respectively. The number of samples in each category and statistical significance are presented in Table 1, Experiment 2.

In order to further challenge the performance of the 4 extraction systems to exclude inhibitors of rRT-PCR, 92 stool samples with levels of inhibition ranging from a single cycle to complete inhibition were evaluated. These samples were selected from among archived stool samples previously tested for enterovirus and MS2 after extraction by QIAamp Viral RNA Mini Kit. A sufficient number of samples with high, intermediate, and low levels of inhibitors were chosen for re-analysis to enable comparison between extraction procedures at each of these levels of inhibition. The results of MS2 inhibition for each sample by extraction protocol is shown in Fig. 1 and summarized by levels of inhibition in Table 1, Experiment 2. The number of samples with inhibitors differed among the four protocols and for some individual samples, the amount of inhibition varied depending on the extraction protocol. Specifically, inhibitors were present in 58 (63.0%), 55 (59.8%), 37 (40.2%) and 30 (32.6%) of the samples prepared by Protocols A, B, C, and D, respectively. Median IQR reduction in Ct levels for protocols A, B, C and D were 11.7 (0 to 34), 7.0 (0 to 30), 4.2 (0 to 29), and 1.3 (0 to 30), respectively. More importantly the number and proportion of samples where inhibition was >6 Ct also varied among the protocols; 44 (47.8%), 29 (31.5%), 14 (15.2%), and 1 (1.1%), for Protocols A, B, C, and D, respectively. The Friedman Test indicated that there was a statistically significant difference in co-extraction of inhibitors of MS2 rRT-PCR among the extraction protocols used, *P*<0.001.

### Is Variation in Recovery of MS2 by each of the Four Extraction Protocols Due to the Presence of Inhibitors or Variation in Extraction Efficiency?

**Figure 2:**
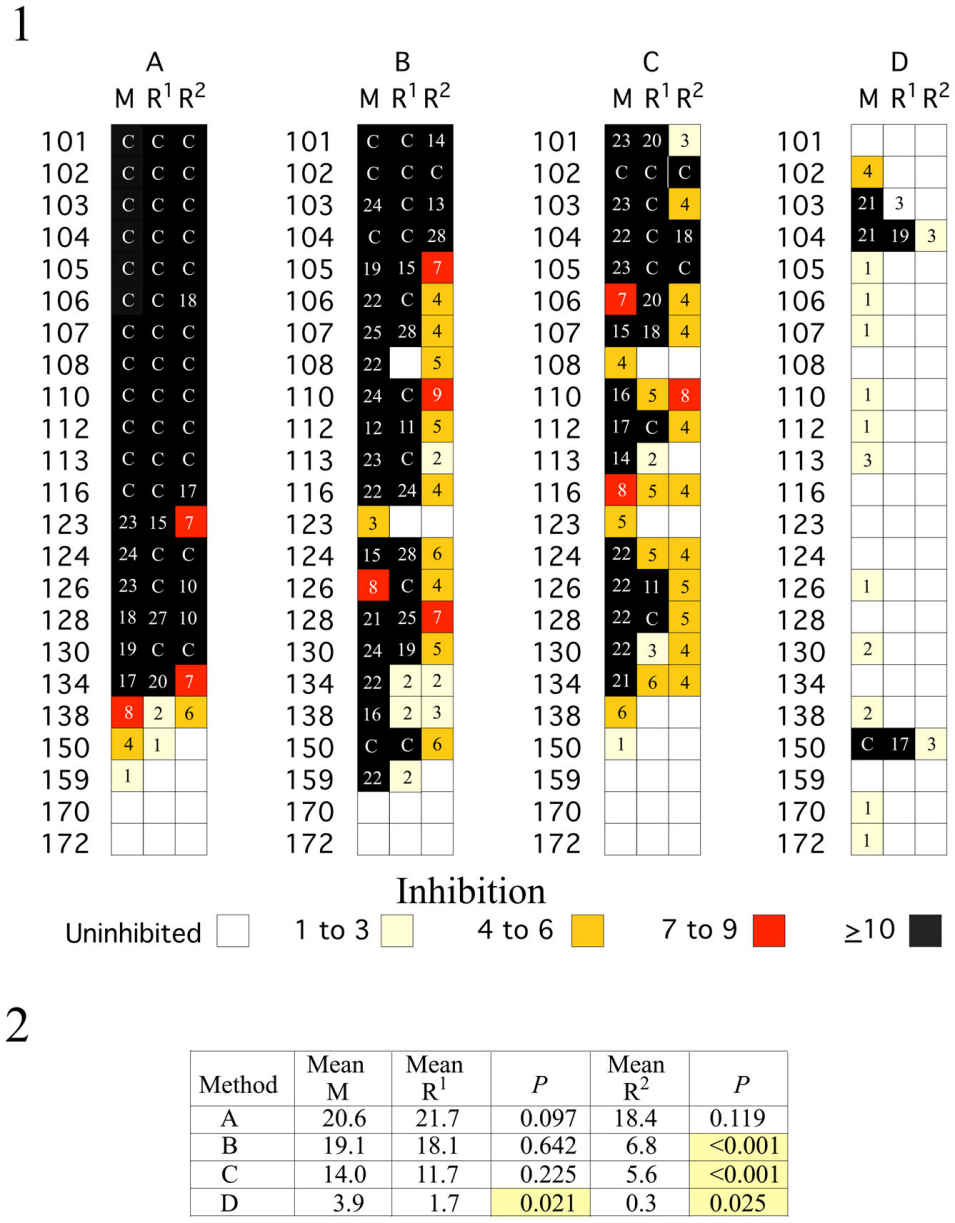
MS2 rRT-PCR inhibition compared with enterovirus rRT-PCR inhibition for enterovirus RNA added after extraction. 1. Two different concentrations of CoxB3 RNA were added to the RNA from 23 stool suspensions after extraction by protocols: (A) QIAgen, (B) MagNA Pure, (C) KingFisher, and (D) easyMag. rRT-PCR values for MS2 RNA extracted from buffer controls or the enterovirus RNA spikes were subtracted from the values for samples from stool suspensions. Results in column R^1^ were obtained when enteroviral RNA (Ct 29) was added to the extracted RNA and those in column R^2^ were obtained when 128 fold (6 CT) more enteroviral RNA was added. The values for inhibition of MS2 rRT-PCR (column M) are the mean of three replicate tests where the RNA was stored at −70°C between each of the MS2 measurements. The individual repeated MS2 measurements are shown in Figure S2. An empty white box indicates that there was no inhibition. Negative values were set to 0 and the maximum values for inhibition “C” were capped at 29 Cts. Samples with inhibition ≥10, 6 to 9, 3 to 6, and 1 to 3 CT are indicated by the colors of the boxes: black, red, tan, and light yellow, respectively. 2. The mean inhibition of MS2 and enterovirus rRT-PCR results is listed for the above by protocol. Yellow boxes indicate the pairwise comparisons where the inhibition of enteroviral rRT-PCR and MS2 differed significantly by Repeated Measurements, Analysis of Variance.

To differentiate between rRT-PCR inhibition due to the presence of inhibitors in the RNA after extraction and variation due to differences in efficiency of extraction, 23 RNA extracts from samples with inhibitors were spiked with two concentrations of purified CoxB3 enterovirus RNA (Fig. 2). The first concentration was equivalent to that of MS2 (R^1^, equivalent) and the second contained 64 fold (6 Cts) more enterovirus RNA (R^2^, high). No significant difference between inhibition of MS2 rRT-PCR and enterovirus rRT-PCR was noted when equivalent amounts of enterovirus RNA was added to extracts prepared by protocols A, B and C (Fig. 2. part 2). However, when the higher amount of enterovirus RNA was added, there was significantly less inhibition of enterovirus rRT-PCR than MS2 rRT-PCR extracts prepared using protocols B, C, and D, but not A. Thus, the decrease in MS2 rRT-PCR can serve as a measure of the amount of inhibitors in the RNA extracted by each of the extraction protocol.

Analysis of variance, indicated that there were no significant differences between inhibition of Cts for MS2 and enterovirus RNA for Protocols A, B, and C, but not D, when the amount of enterovirus RNA was equivalent to that of MS2 (P>0.5), whereas there was significantly less inhibition when the 64-fold higher amount of enteroviral RNA was tested for RNA extracted by protocols B, C, and D (*P*<0.05), but not A (*P*>0.05).

### Comparison of MS2 rRT-PCR and Enterovirus rRT-PCR in Reconstructed Clinical Stool Suspensions Spiked with Intact Virus Before Extraction by Four Different Protocols

**Figure 3:**
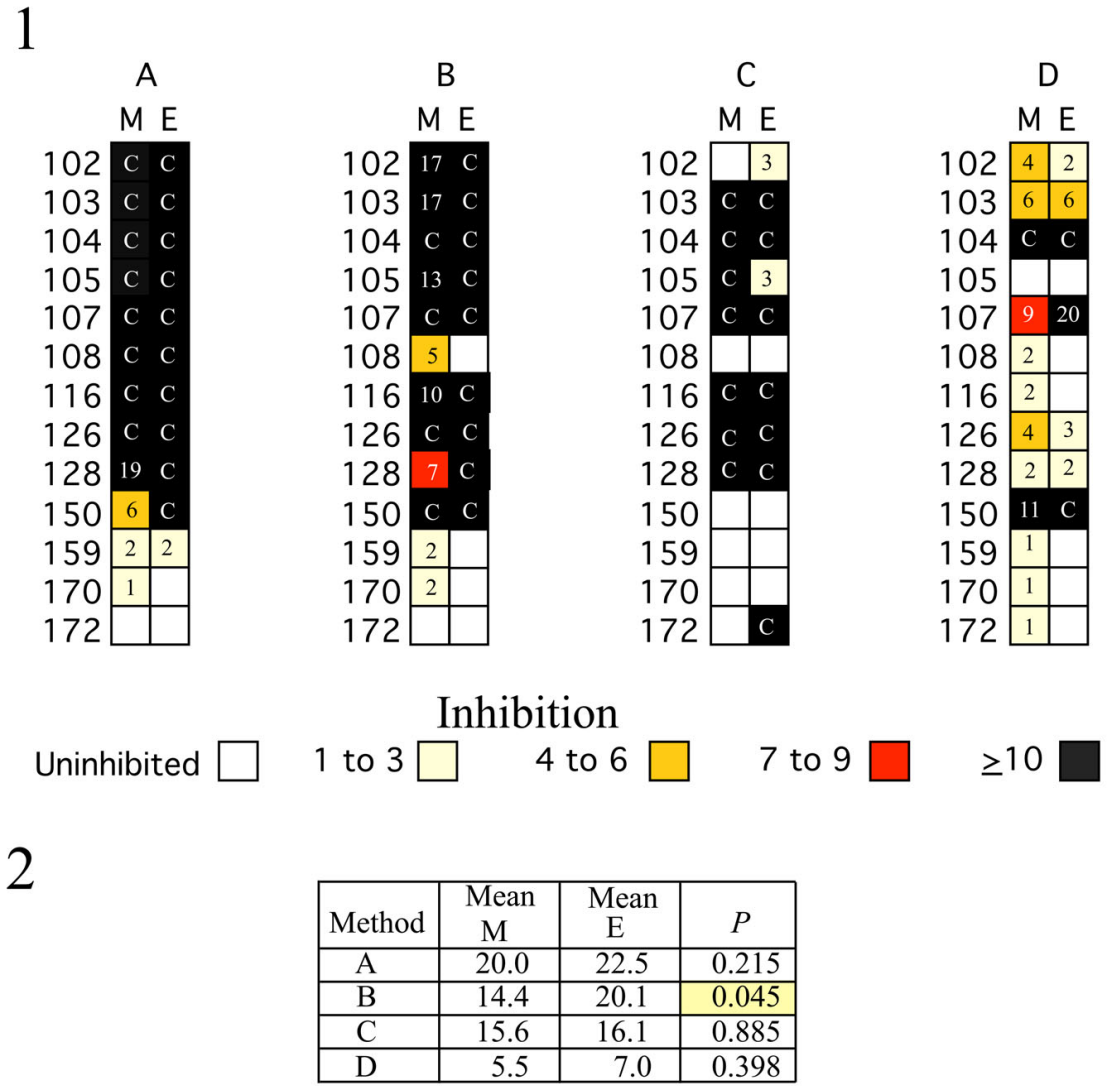
Inhibition of enterovirus and MS2 rRT-PCRs when enterovirus was added before extraction of stool suspensions. 1. CoxB3 virus was added to thirteen of the stool suspensions characterized in the experiment shown in Fig 2. The intact virus was added at a concentration calculated to yield a Ct of 27 and the suspensions were re-extracted by protocols: (A) QIAgen, (B) MagNA Pure, (C) KingFisher, and (D) easyMag as described in Methods. The values for inhibition of MS2 rRT-PCR are in Column M, while those for enterovirus are shown in Column E. An empty white box indicates that there was no inhibition. Negative values were set to 0 and the maximum values for inhibition “C” were capped at 29 Cts. Samples with inhibition ≥10, 6 to 9, 3 to 6, and 1 to 3 CT are indicated by the colors of the boxes: black, red, tan, and light yellow, respectively. 2. The mean inhibition of MS2 and enterovirus rRT-PCR results is listed for the above by protocol. Yellow boxes indicate the pairwise comparisons where the inhibition of enteroviral rRT-PCR and MS2 differed significantly by Repeated Measurements, Analysis of Variance.

Thirteen stool suspensions with varying amounts of inhibitors were spiked with intact CoxB3 virus at a concentration adjusted to yield a Ct equivalent to that of the MS2. These spiked suspensions were re-extracted in parallel by all four methods. With few exceptions, the extent of inhibition of enterovirus rRT-PCR was similar to that for inhibition of MS2 rRT-PCR (Fig. 3). The number of samples with ≥6 Ct inhibition in MS2 rRT-PCR, that also had >6 Ct inhibition of enterovirus rRT-PCR, was 10/10 (100%), 9/9 (100%), 6/7 (85.7%), and 4/4 (100%) for Protocols A, B, C, and D, respectively.

Analysis of variance (Fig. 3, part 2), indicated that there was no significant difference (paired t-test, *P*>0.05) between the inhibition of rRT-PCR of the MS2 external control and the added enterovirus (*P*>0.05) for protocols A, C, and D. In protocol B the inhibition of the rRT-PCR for enterovirus was actually significantly higher than that for MS2 (*P*  = 0.045). Recovery of enterovirus RNA by the four different protocols was equivalent.

## Discussion

One of the trends in pathogen identification is the increasing reliance on molecular methods to achieve high throughput, high sensitivity and specificity of results in clinically relevant times [2], [16]. However, low pathogen copy number, inefficient extraction and the incomplete removal of inhibitors, reduce the sensitivity and specificity of the molecular assays. Among clinical samples, stool suspensions contain a wide range of materials that have the potential to inhibit RT and/or PCR chemistries [4], [6], [8], [9], [10], [11]. Thus, it is important to develop a reliable method to determine whether inhibitors are present in nucleic acid prepared directly from stool suspensions. An appropriate internal control does not exist because of wide differences in eukaryotic and prokaryotic composition in fecal material between patients.

Four protocols for extracting RNA from stool samples were compared in this study. These four commercial extraction methods are among the most commonly used in the diagnostic laboratories. None of samples 1 to 93 contained MS2 RNA. Similarly Nonove et al (14) did not report any unusual amounts of MS2 RNA that would have indicated prior presence of MS2 in their samples when they added MS2 to 106 stool suspensions at concentrations two to eight-fold less than used in this study. MS2 coliphage was thus used as a non-competitive external control because of its absence from human clinical samples. MS2 is a better external control for rRT-PCR than plasmid or phage DNA since it also measures the efficiency of the RT reaction [5], [12], [13], [14], [17], [18]. Here, the MS2 was added to the lysis buffers to minimize sample manipulation and increase the uniformity between manual and semi-automatic extraction protocols. The presence of rRT-PCR inhibitors was evaluated by extracting equal volumes of stool suspension and eluting with equal volumes of elution buffer. Extractions were performed in parallel to eliminate storage related differences, and RNA was assayed on the same rRT-PCR run to minimize analytical differences [19]. Absence of MS2 in the stool samples 1–93 was confirmed by MS2 rRT-PCR of RNA extracted with the EasyMag RNA extraction system using lysis buffer without exogenous MS2. This extraction protocol, the system that had the least amount of samples with inhibitors, was chosen to increase the chance of finding any endogenous MS2 RNA in the stools.

The number of stool suspension RNA extracts with inhibitors of MS2 rRT-PCR varied between RNA extraction protocols for randomly chosen stool suspensions. The amount of inhibitors in RNA extracts for individual stool suspensions also varied according to extraction procedures. This suggests that more than one type of inhibitor may be present and that different procedures do have different proficiencies in excluding them. Stool samples extracted by the KingFisher and the NucliSENS easyMag had fewer extracts with inhibitors than the QIAamp Viral RNA Mini Kit and MagNA Pure LC2.0 Automatic extractor. The results for samples 1 to 93 with the MagNA Pure LC2.0 Automatic extractor were similar to the 21% reported earlier by Ninove et al. [14] using the same protocol on 106 stool samples. A number of other studies [18], [20], [21], [22] found that procedures similar to that used by EasyMag outperformed manual extraction procedures similar to QIAgen, and that MagNA Pure and KingFisher-like procedures were intermediate. In contrast fewer did not find major differences between some of these systems [23], [24]. Results presented here further confirm that RNA extracted with magnetic bead-based systems contained fewer inhibitors than column-based systems [21].

The number of samples with inhibitors was low in the first group of samples (samples 1–93) that were used to compare RNA extraction procedures. In order to better compare the four extraction procedures, we selected from among previously tested samples, sufficient numbers of archived stool suspensions that had high, intermediate, and low levels of inhibitors after extraction by QIAamp Viral RNA Mini Kit (samples 101–192). The stool suspensions were re-extracted in parallel by all four protocols and evaluated for inhibition of MS2 rRT-PCR. As before, KingFisher and NucliSENS EasyMag semi were better than the QIAamp Viral RNA Mini Kit and MagNA Pure LC2.0 Automatic extractor.

Differences in extraction efficiency might be at least as important as inhibitors, as Hata et al. [25] showed by adding separate controls for both extraction and amplification. We performed a similar analysis by adding enterovirus RNA to RNA samples after extraction and intact enterovirus to suspensions before extraction. The inhibition of enterovirus rRT-PCR correlated with MS2 rRT-PCR inhibition. It is important to note that all four extraction protocols yielded equivalent amounts of enterovirus RNA from enterovirus added to suspensions where there was no inhibition of MS2 RNA rRT-PCR in the samples for equal starting and elution volumes. The pattern and amount of enterovirus rRT-PCR inhibition was similar for specific suspensions regardless of whether enterovirus was added before extraction or viral RNA added to the reaction mix after extraction. This implies that the apparent decrease in the amount of MS2 RNA in an RNA extract where MS2 RNA was added to the lysis buffer was primarily due to failure to remove inhibitors present in particular stool suspensions.

The standard maximum volumes that could be extracted differ for the procedures: 140 µl for QIAgen; 100 µl for MagNA Pure; 50 µl for KingFisher; and, 200 µl for easyMag. The effect of using these manufacturers’ recommended volumes for stool suspensions was not evaluated in the present study, although to have done so would have meant that depending on the procedure used, there would have been a 2 to 4 fold increase in the amount of endogenous inhibitors in the starting volume without a concomitant increase in the extraction, washing and elution buffer volumes. Others have shown that increasing the starting volume increased the co-extraction of inhibitors [21], [25], [26] and that the final rRT-PCR outcome is a balance between specific template and coextracted inhibitors [27].

The majority of rRT-PCR assays have a lower limit of quantitation of 10 target sequences per reaction with a Ct of approximately 35 cycles. A reaction with a three-fold shift upwards in Ct due the presence of inhibitors would still be positive, i.e. a specific signal would still be detectable below the 40 cycle cap recommended for the majority of rRT-PCR assays. A two Ct difference is within the statistical variation that may occur between repeated analyses of the same RNA. In contrast, a positive signal would no longer be detectible if there were a six-fold shift in Ct, which would produce a false negative outcome.

In conclusion, inhibition is a complex process. All four extraction methods were suitable provided that an external control was used to identify problematic samples. rRT-PCR of MS2 RNA recovered from MS2 coliphage added to the lysis buffers of RNA extraction systems is a good predictor of inhibition of enteroviral RNA extracted from stool suspensions. More than one inhibitor may be present in the stool suspensions or added during extraction and their efficiency of removal differs between the extraction protocols. The correlation between the extent of MS2 rRT-PCR inhibition and enteroviral rRT-PCR inhibition increases inversely in relation to the amount of enteroviral RNA in the sample. In agreement with Dreier et al [5], MS2 rRT-PCR inhibition should be tested for each RNA sample from a stool suspensions each time it is tested since there is no way to predict in advance whether inhibitors have been efficiently removed during extraction or remain active after cycles of frozen storage. Viral rRT-PCR results should not be considered as quantitative results when MS2 rRT-PCR is inhibited by more than 3 Ct. Finally, we recommend that any negative viral rRT-PCR result from a sample with an inhibition of >6 Ct for MS2 rRT-PCR should be considered invalid and alternative methods used to re-assay or re-extract the sample. If the RNA is diluted and re-tested only samples with positive results for enterovirus rRT-PCR should be considered as valid.

## Materials and Methods

### Ethics Statement

The Ethical Review Board of the Sheba Medical Center, Tel Hashomer approved this study (SMC-8859). The samples and results were stripped of all links to personal details pertaining to, or which could be used to identify individual patients. All data were analyzed anonymously. The Ethical Review Board exempted this study from a requirement for obtaining informed consent.

### Clinical Samples

Stool suspensions (N = 185) prepared for routine analysis of clinical stool samples sent to the Central Virology Laboratory (CVL) at Chaim Sheba Medical Center in Israel were used to evaluate the efficiency of four different RNA extraction systems in excluding inhibitors of rRT-PCR. Ninety-three of the stool samples contained unknown amounts of rRT-PCR inhibitors (Table 1; Fig. S1). The remaining ninety-two stools suspensions were selected from among 860 stool suspensions archived between 2009 and 2011 that had been sent for routine enterovirus analysis (Table 1, Figs. 1, 2, 3 and S2). The RNA from these archived suspensions had been extracted manually with the QIAamp Viral RNA Mini Kit and MS2 rRT-PCR and inhibition levels were known. MS2 rRT-PCR inhibitors >5 Ct were found in 93 (10.8%) of these samples. A non-random subset of 92 of these 860 samples (samples 101 to 192) with high, intermediate, and low levels of inhibitors were chosen for re-analysis so that would be a sufficient number of samples for comparison at each of these levels of inhibition.

### Stool Suspension

Small portions of fecal matter were vortexed for 15 seconds in stool suspension buffer, 2 ml 0.9% saline with glass beads (samples 1 to 93) or 5 ml of M199 containing 15.6 µg of dihydrostreptomycin, 15,625 U of Penicillin G, and 156 U of Mycostatin and 0.1 volume (v/v) of chloroform (samples 101–192). Suspensions were clarified by centrifugation at 2,500×g for 10 min and stored at −20°C until use.

### MS2 Coliphage External Control

A natural *E. coli* RNA MS2 coliphage [28] (MS2, ATCC 15597-B1) stock was prepared on *E. coli Top 10F* in NZYCM broth as described by Dreier et al [5]. Aliquots of the stock were frozen at −70°C. The concentrated stock was thawed, serially diluted in dilution buffer [100 mM NaCl, 8 mM MgSO_4_, 50 mM Tris pH 7.5 and 0.01% (w/v) gelatin] and added to the respective extraction lysis buffers used in each of the extraction procedures before use. The amount of external control MS2 template added to the lysis buffers was adjusted to give a Ct of approximately 27 (∼10,000 copies/ml) in the rRT-PCR mix upon addition of 5 µl of RNA.

### RNA Extraction

RNA was extracted from clarified fecal suspensions using four different commercial protocols. Samples were extracted in parallel to eliminate storage related differences [19]. The four protocols used were: ***Protocol A***, manual extraction - QIAamp Viral RNA Mini Kit (QIAGEN Inc, Valencia, CA, USA), 140 µl aqueous solution extracted and eluted into 50 µl.; ***Protocol B***, automatic RNA extraction - MagNA Pure LC2.0 Automatic extractor with MagNA Pure LC Total Nucleic Acid Isolation kit-High Performance (Roche Diagnostics, IN, USA), 100 µl aqueous solution extracted and eluted into 50 µl; ***Protocol C***, semiautomatic extraction-KingFisher (Thermo Electron Corporation, Waltham, MA, USA), using an Ambion MagMAX Viral RNA Isolation kit (Ambion, Inc, Austin, Tx, USA), 50 µl aqueous solution extracted and eluted into 50 µl.; and ***Protocol D***, semi-automatic extraction-NucliSENS easyMag semi-automatic extractor (bioMerieux, Marcy l’Etoile, France ) using the easyMag extraction kit, 200 µl aqueous solution extracted and eluted into 55 µl. Extractions were performed according to manufacturers’ instructions except that RNA was extracted from 50 µl of stool suspension for all four protocols with addition of respective suspension buffer to reach the recommended aqueous volumes listed above. The RNA was stored at −70°C pending analysis and between analyses (See Fig. S2). Samples 1 to 93 were also extracted by protocol D as above, except that exogenous MS2 was omitted from the lysis buffer.

### Semi-quantitative RealTime RT-PCR (rRT-PCR)

The ABI Prism 7500 sequence detection system (Applied Biosystems, Foster City, CA, USA) was used for the amplification and detection of the MS2 and Enterovirus RNA by TaqMan technology as previously described [5], [29], [30]. Briefly, for MS2 rRT-PCR, 5 µl of RNA was added to the AgPath Mastermix (Ambion, Applied Biosystems Inc, Foster City, California), which contained the published concentrations of primers and probes and 5-carboxy-X-rhodamine succinimidyl ester (ROX) as an internal reference dye, whereas 8 µl of RNA was added for all enterovirus rRT-PCR assays. rRT-PCR was performed under the following conditions: 30 min at 48°C, 10 min at 95°C, and 60 cycles of 15 s at 95°C and 1 min at 60°C. RNAs extracted from the same stool suspension by the four procedures were assayed on the same rRT-PCR run to minimize analytical differences [19].

### Data Management and Analysis

Data were managed and analyzed using Excel (Microsoft) and SPSS (ver. 15) software. For each extraction protocol, the presence and relative amount of rRT-PCR inhibitor(s) was determined by comparing MS2 rRT-PCR Ct results obtained in the absence or presence of stool suspensions. Measured MS2 Cts results were assigned to one of five levels of inhibition: no inhibition, inhibition of 1 to 3 Cts (2 to 8 fold), inhibition of 4 to 6 Cts (16 to 64 fold), inhibition of 7 to 9 Cts (128 to 512 fold) and inhibition of ≥10 Cts (≥1024 fold). Similarly, the presence and relative amount of inhibitors of enterovirus RNA rRT-PCR was determined by comparing enterovirus rRT-PCR Ct results obtained in the absence or presence of stool suspensions for enterovirus added before extraction or enterovirus RNA added after extraction. The inhibition for suspensions that gave Cts for MS2 rRT-PCR below that for MS2 in suspension buffer alone was set to 0. The upper limit for rRT-PCR inhibition was capped at 29 Ct.

The non-parametric Friedman Test was used to determine to determine whether there were significant differences between results among the four different extraction protocols. Post-hoc analysis of pairwise differences between protocols was performed using a Wilcoxon Signed-Rank Test (SPSS) with a Bonferroni correction that set the significance level for the pairwise comparisons to P<0.008. Comparison of MS2 rRT-PCR and enteroviral rRT-PCR Cts was by repeated measurements, analysis of variants.

## Supporting Information

Figure S1 **MS2 rRT-PCR inhibition in RNA extracted from stool suspensions using four different RNA extraction protocols.** Equal amounts of stool suspensions chosen randomly from among samples sent to the laboratory were extracted by four protocols: (A) QIAgen, (B) magNA Pure, (C) KingFisher, and (D) easyMag as described in Methods. MS2 coliphage calculated to give 27 Ct by rRT-PCR was added to the extraction buffer. rRT-PCR values for MS2 in RNA extracted from buffer controls were subtracted from the values for MS2 in RNA extracted from stool suspensions. This difference, the number of Cts of inhibition, is shown in the box to the right of the sample numbers. Negative values were set to 0 and the maximum values for inhibition “C” were capped at 29 Cts. Samples with inhibition >10, 7 to 9, 4 to 6, and 1 to 3 Ct are indicated by the colors of the boxes: black, red, tan, and light yellow, respectively. Blank white boxes indicate no inhibition. The numbers of samples in each category and the significance in differences are shown in Table 1, Experiment 1.
(PDF)

Figure S2 **MS2 rRT-PCR inhibition for RNA extracted from stool after re-freezing and thawing by extraction protocol. 1.** Three repeated measurements of MS2 rRT-PCR were performed for the 23 samples by protocols: (A) QIAgen, (B) magNA Pure, (C) KingFisher, and (D) easyMag as described in Methods. The RNA was stored at −70°C between tests. MS2 coliphage was added to the extraction buffer. rRT-PCR values for MS2 in RNA extracted from buffer alone were subtracted from the values for MS2 in RNA extracted from stool suspensions. This difference, the number of Cts of inhibition, is shown in the boxes to the right of the sample numbers. Negative values were set to 0 and the maximum values for inhibition “C” were capped at 29 Cts. Samples with inhibition ≥10, 7 to 9, 4 to 6, and 1 to 3 Ct are indicated by the colors of the boxes: black, red, tan, and light yellow, respectively. Blank white boxes indicate that the samples were not inhibited. Samples were stored at −70°C between measurements. 2. The means for each of the three repeat tests the square of the means (M Sq), the standard deviation of each run (S.D.), and the significance of differences between the means of the repeated measurements by extraction protocol are presented. There were no significant differences between the repeat measurements for each extraction protocol. 3. Friedman test values for comparison among the 4 extraction methods. There were significant differences among the extraction protocols (shaded yellow boxes). 4. The Wilcoxon Singed-Rank Test (SPSS) with Bonferroni correction (Significance if P<0.008) for the pair-wise comparison of the means of the three measurements of the rRT-PCR results from the different extraction protocols. Shaded yellow boxes indicate where pairwise differences between extraction protocols were significant.
(PDF)
