# Supplementary figures and images for: Evaluation of Four Different Systems for Extraction of RNA from Stool Suspensions Using MS-2 Coliphage as an Exogenous Control for RT-PCR Inhibition
Source: PLoS One. 2012 Jul 16;7(7):e39455. doi: 10.1371/journal.pone.0039455 (PMC3397973; doi:10.1371/journal.pone.0039455)

|    | A | B  | C | D |  | A  | B | C | D  |   | A  | B | C | D |  | A  | B | C | D |  | A  | B | C | D |  | A | B | C | D |
|----|---|----|---|---|--|----|---|---|----|---|----|---|---|---|--|----|---|---|---|--|----|---|---|---|--|---|---|---|---|
| 1  | C | C  | C | 3 |  | 21 |   | 5 |    |   | 41 |   |   |   |  | 61 |   |   |   |  | 81 |   |   |   |  |   |   |   |   |
| 2  | C | C  | 4 | 3 |  | 22 |   | 5 |    |   | 42 |   |   |   |  | 62 |   |   |   |  | 82 |   |   |   |  |   |   |   |   |
| 3  | C | 3  |   |   |  | 23 |   | 4 | 2  | 7 | 43 |   |   |   |  | 63 |   |   |   |  | 83 |   |   |   |  |   |   |   |   |
| 4  | C | 1  |   |   |  | 24 |   | 4 |    |   | 44 |   |   |   |  | 64 |   |   |   |  | 84 |   |   |   |  |   |   |   |   |
| 5  | C |    |   |   |  | 25 |   | 4 |    |   | 45 |   |   |   |  | 65 |   |   |   |  | 85 |   |   |   |  |   |   |   |   |
| 6  | C |    |   |   |  | 26 |   | 3 |    |   | 46 |   |   |   |  | 66 |   |   |   |  | 86 |   |   |   |  |   |   |   |   |
| 7  | 6 |    |   |   |  | 27 |   | 3 |    |   | 47 |   |   |   |  | 67 |   |   |   |  | 87 |   |   |   |  |   |   |   |   |
| 8  | 3 | 7  |   | 3 |  | 28 |   | 2 |    |   | 48 |   |   |   |  | 68 |   |   |   |  | 88 |   |   |   |  |   |   |   |   |
| 9  | 3 | 3  |   |   |  | 29 |   | 1 |    |   | 49 |   |   |   |  | 69 |   |   |   |  | 89 |   |   |   |  |   |   |   |   |
| 10 | 3 | 2  |   |   |  | 30 |   |   | 11 |   | 50 |   |   |   |  | 70 |   |   |   |  | 90 |   |   |   |  |   |   |   |   |
| 11 | 3 | 2  |   |   |  | 31 |   |   | 3  |   | 51 |   |   |   |  | 71 |   |   |   |  | 91 |   |   |   |  |   |   |   |   |
| 12 | 2 | 4  |   | 3 |  | 32 |   |   | 2  |   | 52 |   |   |   |  | 72 |   |   |   |  | 92 |   |   |   |  |   |   |   |   |
| 13 |   | 10 |   |   |  | 33 |   |   | 2  |   | 53 |   |   |   |  | 73 |   |   |   |  | 93 |   |   |   |  |   |   |   |   |
| 14 |   | 9  |   |   |  | 34 |   |   | 1  |   | 54 |   |   | 2 |  | 74 |   |   |   |  |    |   |   |   |  |   |   |   |   |
| 15 |   | 9  |   |   |  | 35 |   |   |    |   | 55 |   |   |   |  | 75 |   |   |   |  |    |   |   |   |  |   |   |   |   |
| 16 |   | 6  |   |   |  | 36 |   |   |    |   | 56 |   |   |   |  | 76 |   |   |   |  |    |   |   |   |  |   |   |   |   |
| 17 |   | 6  |   |   |  | 37 |   |   |    |   | 57 |   |   |   |  | 77 |   |   |   |  |    |   |   |   |  |   |   |   |   |
| 18 |   | 6  |   |   |  | 38 |   |   |    |   | 58 |   |   |   |  | 78 |   |   |   |  |    |   |   |   |  |   |   |   |   |
| 19 |   | 6  |   |   |  | 39 |   |   |    |   | 59 |   |   | 3 |  | 79 |   |   |   |  |    |   |   |   |  |   |   |   |   |
| 20 |   | 6  |   |   |  | 40 |   |   |    |   | 60 |   |   |   |  | 80 |   |   |   |  |    |   |   |   |  |   |   |   |   |

Inhibition

$\geq 10$  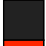  
 7 to 9 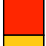  
 4 to 6 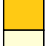  
 1 to 3 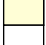  
 uninhibited 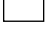

Supplement: Figure S1 — MS2 rRT-PCR inhibition in RNA extracted from stool suspensions using four different RNA extraction protocols. Equal amounts of stool suspensions chosen randomly from among samples sent to the laboratory were extracted by four protocols: (A) QIAgen, (B) magNA Pure, (C) KingFisher, and (D) easyMag as described in Methods. MS2 coliphage calculated to give 27 Ct by rRT-PCR was added to the extraction buffer. rRT-PCR values for MS2 in RNA extracted from buffer controls were subtracted from the values for MS2 in RNA extracted from stool suspensions. This difference, the number of Cts of inhibition, is shown in the box to the right of the sample numbers. Negative values were set to 0 and the maximum values for inhibition “C” were capped at 29 Cts. Samples with inhibition >10, 7 to 9, 4 to 6, and 1 to 3 Ct are indicated by the colors of the boxes: black, red, tan, and light yellow, respectively. Blank white boxes indicate no inhibition. The numbers of samples in each category and the significance in differences are shown in Table 1, Experiment 1. (PDF) [file pone.0039455.s001.pdf]
