# Supplementary material for: Evaluation of Four Different Systems for Extraction of RNA from Stool Suspensions Using MS-2 Coliphage as an Exogenous Control for RT-PCR Inhibition
Source: PLoS One. 2012 Jul 16;7(7):e39455. doi: 10.1371/journal.pone.0039455 (PMC3397973; doi:10.1371/journal.pone.0039455)
Supplement: Figure S2 — MS2 rRT-PCR inhibition for RNA extracted from stool after re-freezing and thawing by extraction protocol. 1. Three repeated measurements of MS2 rRT-PCR were performed for the 23 samples by protocols: (A) QIAgen, (B) magNA Pure, (C) KingFisher, and (D) easyMag as described in Methods. The RNA was stored at −70°C between tests. MS2 coliphage was added to the extraction buffer. rRT-PCR values for MS2 in RNA extracted from buffer alone were subtracted from the values for MS2 in RNA extracted from stool suspensions. This difference, the number of Cts of inhibition, is shown in the boxes to the right of the sample numbers. Negative values were set to 0 and the maximum values for inhibition “C” were capped at 29 Cts. Samples with inhibition ≥10, 7 to 9, 4 to 6, and 1 to 3 Ct are indicated by the colors of the boxes: black, red, tan, and light yellow, respectively. Blank white boxes indicate that the samples were not inhibited. Samples were stored at −70°C between measurements. 2. The means for each of the three repeat tests the square of the means (M Sq), the standard deviation of each run (S.D.), and the significance of differences between the means of the repeated measurements by extraction protocol are presented. There were no significant differences between the repeat measurements for each extraction protocol. 3. Friedman test values for comparison among the 4 extraction methods. There were significant differences among the extraction protocols (shaded yellow boxes). 4. The Wilcoxon Singed-Rank Test (SPSS) with Bonferroni correction (Significance if P<0.008) for the pair-wise comparison of the means of the three measurements of the rRT-PCR results from the different extraction protocols. Shaded yellow boxes indicate where pairwise differences between extraction protocols were significant. (PDF) [file pone.0039455.s002.pdf]

1

	A1	A2	A3	B1	B2	B3	C1	C2	C3	D1	D2	D3
101	C	C	C	C	C	C	C	17	24			1
102	C	C	C	C	C	C	C	C	C	4	3	5
103	C	C	C	C	15	C	C	10	C	5	C	C
104	C	C	C	C	C	C	14	23	C	5	C	C
105	C	C	C	C	10	18	12	C	C			2
106	C	C	C	C	9	C	7	6	8			2
107	C	C	C	C	C	17	7	C	9	2		2
108	C	C	C	C	9	C	3	2	6			1
110	C	C	C	15	C	C	11	C	8	2		2
112	C	C	C	10	8	18	C	11	10	2		2
113	C	C	C	10	C	C	C	5	8	4		5
116	C	29	C	8	C	C	6	8	9			1
123	24	25	20	3	4	3	2	5	9			1
124	23	21	C	C	10	7	C	7	C			
126	20	19	C	6	8	9	C	7	C	2		3
128	14	12	C	C	C	6	8	C	C			
130	13	25	18	C	15	C	C	7	C	2		
134	10	12	C	7	C	C	6	C	C			1
138	9	9	6	10	C	8	6	7	6	3		3
150	3	5	3	C	C	C			3	C	C	C
159		2	2	C	7	C			1			
170			1			1			1			2
172									1			2

Inhibition

uninhibited | 1 to 3 | 4 to 6 | 7 to 9 | ≥10

2

Method	Mean First	Mean Second	Mean Third	M Sq	S.D.	*P*
A	20.2	20.8	22.3	28.9	3.46	0.101
B	19.4	18.0	20.2	27.9	8.65	0.691
C	13.7	12.6	15.8	63.4	8.64	0.434
D	2.6	3.9	5.3	41.8	4.09	0.094

3

	A	B	C	D	*P*
mean	21.1	19.2	14.0	3.9	<0.001
mean ranks	1.74	2.74	3.26	2.26	<0.001

4

	B	C	D
A	0.001	<0.001	0.909
B		0.543	0.017
C			0.006
